# Incorporation of human–wildlife interactions in ecosystem‐based management to enhance conservation of endangered guitarfish

**DOI:** 10.1111/cobi.14327

**Published:** 2024-07-11

**Authors:** Yaara Grossmark, Barak Azriali Zohar, Adi Barash, Michelle E. Portman

**Affiliations:** ^1^ MarCoast Ecosystems Integration Lab Technion – Israel Institute of Technology Haifa Israel; ^2^ Sharks in Israel (NGO) Kibbutz Amir Israel

**Keywords:** citizen science, conservation planning, ecosystem‐based management, human–wildlife interaction, marine planning, spatial conservation prioritization, ciencia ciudadana, interacción humano‐fauna, gestión basada en el ecosistema, planeación de la conservación, planeación marina, priorización espacial de la conservación, 保护规划, 海洋规划, 人类与野生动物互动, 公民科学, 空间优先保护化, 基于生态系统的管理

## Abstract

Growing human use of the marine environment increases the proximity of humans to marine wildlife and thus likely increases human–wildlife interactions. Such interactions influence perceptions of nature and promote or undermine conservation. Despite their importance, human–wildlife interactions are rarely considered in ecosystem‐based marine spatial planning (MSP). Ideally, these interactions should be identified and considered in ecosystem‐based management (EBM), which is often purported to be the basis for MSP. We used Marxan software and data from a citizen science project documenting location, species, age, sex, and activity type to identify regions along Israel's coast with a high probability of encounters between people and 2 species of guitarfish. We considered the geographic distribution of these encounters and the various activities undertaken by the reporting observers. We ran 4 scenarios in Marxan. Two had conservation goals of 30% and 50% guitarfish habitat protection. In the third and fourth scenarios, we added a 50% conservation goal of human leisure activities to each guitarfish conservation goal. We also conducted a gap analysis between our guitarfish conservation goals and the Israel Nature and Parks Authority's master plan for marine protected areas. We found the park authority was close to meeting the 30% goal but was far from meeting the conservation goal of 50% of guitarfish habitat conservation. Different human uses were more likely to interact with different life stages of guitarfish, and different recreational activities occurred in different areas. Identifying areas of specific human use showed which activities should be addressed in conservation management decisions. Our addition of certain recreational uses to the model of habitat conservation showed how enhancing human dimensions in conservation planning can lead to more holistic ecosystem‐based conservation necessary for effective marine planning.

## Abstract (es)

Incorporación de las interacciones humano‐fauna dentro de la gestión basada en el ecosistema para mejorar la conservación del pez guitarra en peligro

**Resumen:**

El uso creciente que el humano le da al mar incrementa la cercanía de las personas con la fauna marina, lo que probablemente incrementa las interacciones humano‐fauna. Dichas interacciones influyen sobre las percepciones que se tienen de la naturaleza y promueven debilitan la conservación. A pesar de su importancia, pocas veces se consideran las relaciones humano‐fauna dentro de la planeación espacial basada en ecosistemas marinos (PEM). Lo ideal debería ser la identificación y consideración de estas interacciones dentro de la gestión basada en el ecosistema (GBE), la cual con frecuencia se considera como la base de la PEM. Usamos software Marxan y datos de un proyecto de ciencia ciudadana que documenta la ubicación, especie, edad, sexo y tipo de actividad para identificar las regiones de la costa de Israel con una alta probabilidad de encuentros entre las personas y dos especies de pez guitarra. Consideramos la distribución geográfica de estos encuentros y las diferentes actividades que realizan los observadores. En Marxan corrimos cuatro escenarios. Dos de los escenarios contaban objetivos de conservación del 30% y 50% de la protección del hábitat del pez guitarra. En los otros dos escenarios, añadimos un objetivo de conservación de 50% de las actividades humanas de recreación a los objetivos uno y dos. También realizamos un análisis de brecha entre los objetivos de conservación del pez guitarra y el plan maestro para las áreas marinas protegidas de la Autoridad de Parques y Naturaleza de Israel. Descubrimos que esta autoridad estaba cerca de lograr el objetivo del 30% pero lejos del de 50% de la conservación del hábitat del pez guitarra. Fue más probable que los diferentes usos humanos interactuaran con diferentes estadios de vida del pez guitarra y las diversas actividades recreativas ocurrieron en áreas distintas. La identificación de las áreas con un uso humano específico mostró cuáles actividades deberían abordarse en las decisiones de gestión de la conservación. La suma de ciertos usos recreativos al modelo de conservación del hábitat mostró cómo aumentar las dimensiones humanas en la planeación de la conservación puede derivar en una conservación basada en el ecosistema más holística, necesaria para la planeación marina eficiente.

## 将人类与野生动物互动纳入基于生态系统的管理, 以加强对濒危犁头鳐的保护

人类对海洋环境的利用日益增加, 导致人类与海洋野生动物的距离越来越近, 这可能加强了人类与野生动物之间的互动。这种互动会影响人们对自然的看法, 从而促进或破坏自然保护。尽管人类与野生动物的互动非常重要, 但在基于生态系统的海洋空间规划中却很少考虑到这一点。基于生态系统的管理常常被认为是海洋空间规划的基础, 理想情况下, 这些管理措施应识别并考虑这些互动。本研究利用Marxan软件和一项公民科学项目中记录的位置、物种、年龄、性别和活动类型等数据, 确定了人类与两种犁头鳐在以色列沿海相遇概率较高的区域。我们考虑了这些相遇事件的地理分布以及报告观察者所进行的各种活动。我们在Marxan软件中模拟了四种情景, 其中两种情景分别设立了保护犁头鳐30%和50%栖息地的保护目标, 而在第三和第四种情景中, 我们在每个犁头鳐保护目标中增加了50%的人类娱乐活动的保护目标。我们还对犁头鳐保护目标与以色列自然和公园管理局的海洋保护区总体规划之间的差距进行了分析。我们发现, 公园管理局已接近实现30%的目标, 但离实现50%的犁头鳐栖息地保护目标还相差甚远。不同人类利用行为更有可能影响犁头鳐的不同生命阶段, 且不同娱乐活动发生在不同的区域。确定特定的人类利用区域指出了保护管理决策应重点关注的人类活动。本研究在栖息地保护模型中纳入了一些人类娱乐活动, 展现了如何在保护规划中增加人类维度以制定更全面的基于生态系统的保护措施, 而这正是有效的海洋规划所必需的。**【翻译:胡怡思;审校:聂永刚】**

## INTRODUCTION

Encounters between humans and wild animals affect people's feelings about nature and thus advance or undermine conservation. Increased activity in the marine environment leads to more frequent encounters with wildlife. These are rarely considered (Niella et al., 2021), despite calls to implement ecosystem‐based management (EBM) as part of marine spatial planning (MSP) processes (Ansong et al., 2017; Ehler & Douvere, 2009a; Irish, 2018; Portman, 2016). EBM considers both ecosystem functioning and human needs to support and enhance ecosystem services (Slocombe, 1998; Szaro et al., 1998). Although many plans claim to be ecosystem based, operationalizing EBM is very difficult (Clark et al., 2022; Cormier et al., 2017; Long et al., 2015; O'Higgins et al., 2020).

EBM seeks to protect and enhance various aspects of the marine and terrestrial environment, including biodiversity, ecosystem function, and ecosystem services. Humans are an integral part of the environment and therefore so are the social dimensions of various activities (Slocombe, 1998). Adaptive management that builds on monitoring data and scientific knowledge is a key principle of EBM (Piet et al., 2020). Hence, a constant stream of spatial, temporal, biological, and socioeconomic data to inform decision‐making (NOAA Science Advisory Board, 2018) is needed. Social media data on human–wildlife interactions can help map the distribution of wildlife and the types of encounters, thus helping to bring the human dimension to EBM decisions.

A human–wildlife interaction is any situation in which a person or their property comes into direct or indirect contact with wildlife (Frank et al., 2019; Nyhus, 2016). All such interactions are on a spectrum between conflict and coexistence (Bhatia et al., 2020; Frank, 2016; Frank & Glikman, 2019); they can stimulate extreme emotions (Conover & Conover, 2021). Conflicts between people and wildlife threaten both humans (Bhatia et al., 2020; König et al., 2020) and wildlife (König et al., 2020; Nyhus, 2016). They invoke public fear and increase misconceptions of risk (Conover & Conover, 2021) of physical harm. Encounters can also be welcomed, as evidenced by popular wildlife viewing trips (Gallagher et al., 2015; Gray et al., 2022; Mangachena & Pickering, 2023). Perhaps most importantly, positive encounters can increase support for biodiversity and species conservation (Marchini et al., 2019; Soulsbury & White, 2019).

How can planners decrease conflicts and increase positive human–wildlife interactions? In general, research on measures to reduce human–terrestrial wildlife encounters exists. Examples include studying the use of wildlife‐crossing structures over busy roads (Glista et al., 2009) and methods to reduce wildlife crop raiding (King, 2019). Zoning has been proposed to mitigate conflicts with large terrestrial carnivores (Linnell et al., 2009). Ocean zoning has also been researched (Agardy, 2010), but it has yet to be pursued as a means of avoiding or promoting human–marine wildlife interactions.

Although interactions are on the rise in the marine environment as a result of old uses (e.g., fishing, mariculture, shipping) and new ones (e.g., energy production, desalination plants), research on human–marine wildlife interactions is limited (Crawford, et al., 2022). Such research has focused mainly on conflicts of marine life with fishers (Guerra, 2019), though some research has incorporated data on ecotourism (Carlucci et al., 2021). Data on such encounters are important for research purposes and for planning praxis. Considering all types of human–wildlife interactions, both positive and negative, will improve the chances of realizing ecosystem‐based MSP.

Data limitations are indeed a problem. Madden (2004) reports that information about negative encounters is often unavailable due to mistrust between the people involved and the authorities. Positive and neutral interactions, despite being more common, are less understood, reported, and addressed (Soulsbury & White, 2019) and are not well defined. Using citizen science to inform decisions about human–wildlife interactions can significantly help with both these situations, especially in the marine environment. Citizen science programs often offer quicker and more cost‐efficient methods of data collection than traditional survey methods, particularly in the marine environment (Giovos et al., 2019; Wichmann et al., 2022).

Citizen science also raises public awareness about and sympathy for research endeavors (Bonney et al., 2016; McAteer & Flannery, 2022; Wichmann et al., 2022). Projects that involve local stakeholders in collecting data help build trust and promote public engagement (Kelly et al., 2020), thus connecting the local community to the planning effort, which is key to successful EBM (Pomeroy & Douvere, 2008). Citizen science can also inform planners and managers about human–wildlife interactions and outcomes of mitigation initiatives over time (Heathcote et al., 2019).

Citizen science data have previously been used in decision‐support tools (Bonnet‐Lebrun et al., 2020; Giovos et al., 2018). We analyzed data on human–wildlife interactions and the distribution of such interactions. We sought to generate a more accurate EBM plan for MSP through a case study of 2 cartilaginous fish species: blackchin guitarfish (*Glaucostegus cemiculus*) and common guitarfish (*Rhinobatos rhinobatos*). Both species are listed as critically endangered by the International Union for Conservation of Nature (IUCN) (Jabado et al., 2021; Kyne & Jabado, 2019). The information on both species is limited (Giovos et al., 2018), yet they are important in soft‐sediment ecosystems because both are mesopredators and prey (Moore, 2017).

A small number of cartilaginous fish studies have been conducted off the coast of Israel (Arial & Barash, 2015), but few are on guitarfish. Chaikin et al. (2020) researched an aggregation of batoids, including the blackchin guitarfish, during spring and early summer in the Gdor Nature Reserve; Azrieli (2020) described 2 areas suspected to be nursery areas for the blackchin guitarfish, of which only one is in a nature reserve. Though thought to be the dominant species, no studies exist on the common guitarfish in Israeli waters, where ecological knowledge on both species is lacking (Azrieli, 2020).

Until 2013, the main threat to the cartilaginous fishes on the Mediterranean coast of Israel was commercial fishing (Arial & Barash, 2015). Cartilaginous fishes comprise about 1.5% of the total Mediterranean Sea commercial landings in Israel, yet stock estimates of the overall cartilaginous fish population in Israel do not exist (Arial & Barash, 2015). Since 2005, all cartilaginous fishes, including guitarfish, have been protected by law (Arial & Barash, 2015; Ministry of Justice, 2019), that is, they are not to be targeted in fishing, if caught as bycatch they are to be released, and they are not to be harassed. Since 2013, protection has been well enforced (Azrieli, 2020).

We aimed to highlight how including data on potential human–wildlife interactions in conservation area optimization modeling can lead to reduced human–wildlife conflict and increased opportunities for conservation. Overall, addressing human–wildlife interactions can advance EBM. We also aimed to show how such data can be gathered, processed, and adapted for use in Marxan to provide a basis for the spatial allocation of uses in the sea, which can inform marine conservation plans and legislation.

## METHODS

We identified areas with a high probability of encounters between people and 2 species of guitarfish along the coast of Israel. In addition to the distribution of encounters, we also considered the different activities undertaken by those documenting it. We identified areas with potential for positive encounter outcomes, such as diving, snorkeling, swimming, and beach walking, and with potential for negative outcomes, such as fishing. The former could benefit from nature conservation measures, such as protection of these areas and promoting sustainable use of guitarfish for ecotourism. Without careful management, even these activities can have negative impacts on guitarfish.

Areas of encounters between guitarfish and fishers were generally of 2 types: angling and free‐dive spearfishing. Recreational angling refers to nonselective fishing with rods from shore. In contrast, free‐dive spearfishing is highly selective, which allows fishers to avoid harming guitarfish.

We used the optimization software Marxan to help identify areas of different recreational activities by considering the activities’ conservation goals. Marxan identifies areas that should be a priority for conservation given certain constraints. Each Marxan run consisted of 100 repetitions that produced a frequency selection map. The frequency of the selection of a particular cell indicated that cell's importance for the set of conservation goals. Subsequently, managers can adapt a conservation plan for these activities. For example, because defining recreational fishing areas as either no entry or no take can arouse angler community opposition, other forms of conservation should be used (e.g., dedicated educational programs for fishers or the prohibition of certain gear types).

### Case study

**FIGURE 1:**
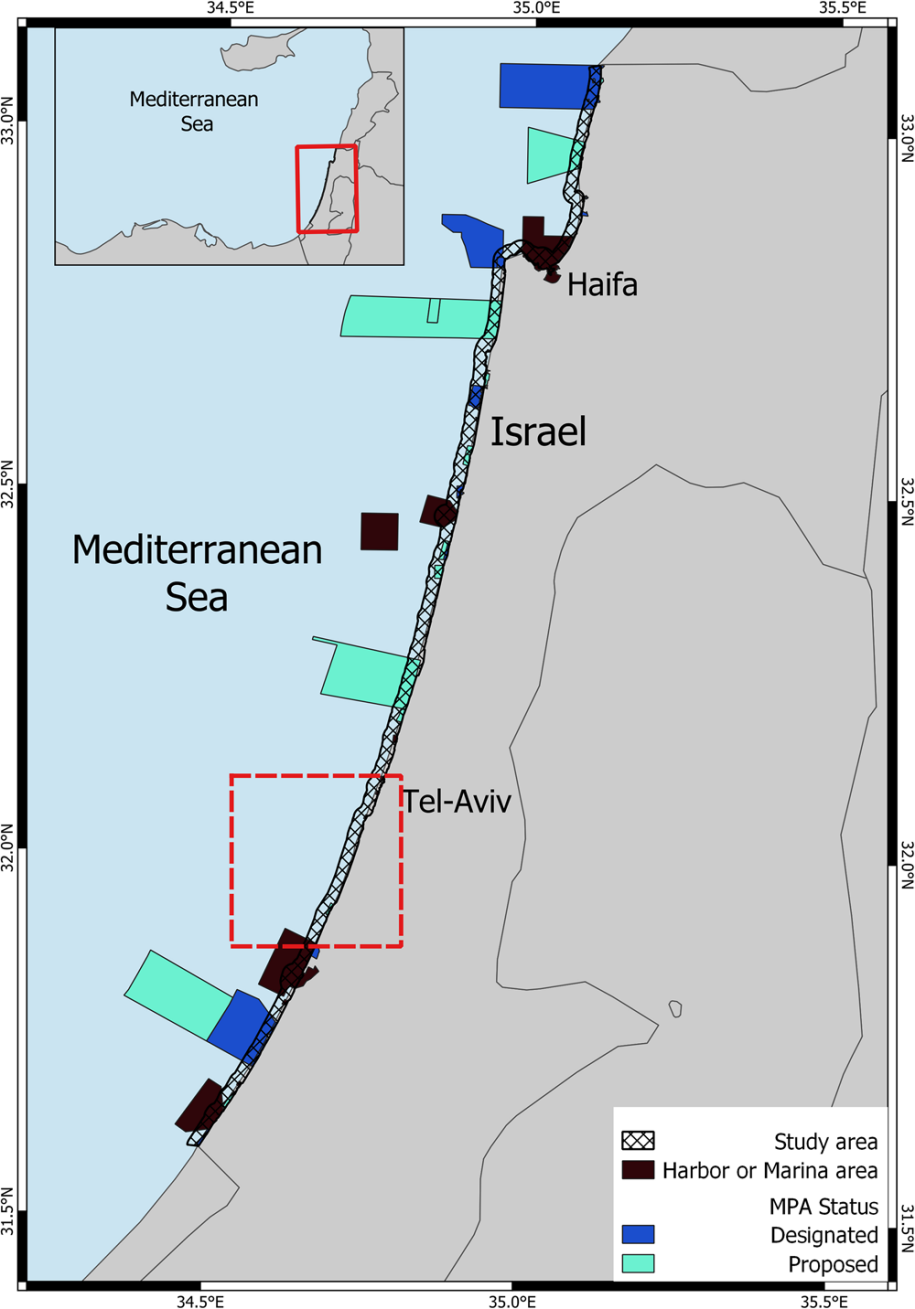
The study area (259 km^2^) showing harbors, marinas, and marine protected areas (MPAs) in Israel and their status (red dashed square, area enlarged in Figures 2, 3, 4 is 36.5 km^2^ [14% of study area]).

Two species of guitarfish populate Israel's Mediterranean coast (Figure 1), the blackchin guitarfish and the common guitarfish (Azrieli, 2020; Barash et al., 2018; Chaikin et al., 2020). According to the IUCN Red List of Threatened Species, both species are critically endangered. They are listed in Annex II of the Specially Protected Areas and Biological Diversity Protocol for the Mediterranean under the Barcelona Convention (Jabado et al., 2021; Kyne & Jabado, 2019) and in CITES (the Convention on International Trade in Endangered Species of Wild Fauna and Flora) Appendix II (https://cites.org/eng). The common guitarfish is also listed in the Convention on the Conservation of Migratory Species of Wild Animals, Appendix II (https://www.cms.int/). Therefore, these 2 species’ conservation is of high priority (Jabado et al., 2021; Kyne & Jabado, 2019).

The 2 species inhabit coastal and estuarine habitats with muddy or sandy bottoms, ranging from very shallow waters up to about 100‐m depth (Newell, 2017). Thus, both species of guitarfish are highly exposed to encounters with people and to the effects of human activities and uses on their surroundings. Moreover, even though the 2 fish species grow to an impressive size, 2 m for the blackchin guitarfish and 1.5 m for the common guitarfish (Ebert & Dando, 2020), they pose no danger to humans. The combination of their large size, harmlessness to people, and accessibility makes them suitable as a tourist attraction for divers and snorkelers. Moreover, the blackchin guitarfish pups stay in very shallow waters, allowing nondivers to observe them (Azrieli, 2020). However, these traits also expose them to unfavorable interactions with fishers.

Although both guitarfish species have been legally protected in Israel since 2005 (Arial & Barash, 2015; Ministry of Justice, 2019), they are still caught as bycatch. When caught, fishers must release the fish back into the sea. Catch and release can negatively affect fish survival, especially if done incorrectly (Gallagher et al., 2017). These adverse effects can be significantly reduced by using appropriate equipment and by implementing correct release practices (Skov et al., 2023).

Our research area was 259 km^2^. The area's borders were chosen with respect to the spatial extent of our data. The data were collected by beachgoers and those engaging in recreational activities close to shore. Just over 13% of the research area is marine protected area (MPA). These MPAs fall under one of 2 legal definitions: “nature reserve” (conservation emphasis) or “national park” (focus on public recreation or historical, architectural, natural, or scenic significance). In the latter areas, nature conservation is a secondary benefit (Law on National Parks, Nature Reserves, National Sites & Memorial Sites, 1998).

**FIGURE 2:**
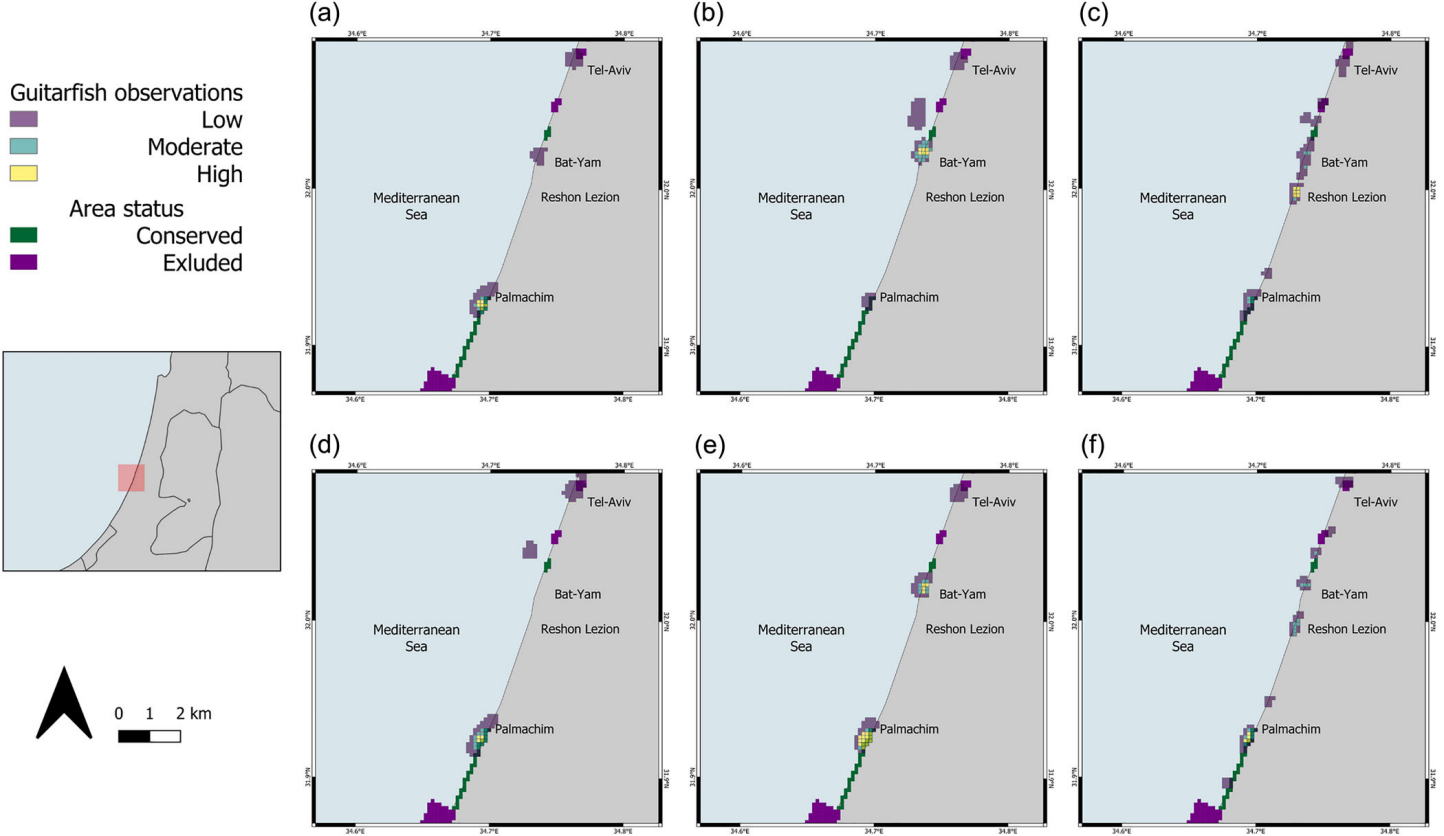
Distributions of observations of (a) adult female blackchin guitarfish, (b) adult male blackchin guitarfish male, (c) juvenile blackchin guitarfish, (d) adult female common guitarfish females, (e) adult male common guitarfish males, and (f) juvenile common guitarfish (percentage of the same guitarfish category observed: low, ≤0.5%; moderate, 0.5–1%; high, >1%).

Most of the smaller MPAs were established before the early 2000s. In 2012, the Israel Nature and Parks Authority (INPA) issued a policy document for nature conservation in the Mediterranean (Yahel & Engert, 2012), following which the authority began the process of declaring 6 large marine nature reserves. Three of these have already been legally approved, and the other 3 are in various stages of planning. Our analyses pertain to only the nearshore areas of these 3 large MPAs (Figure 2). Marinas and ports, covering 18.5% of the study area, were excluded from the analyses (Figure 2). Marinas and port areas are generally highly disturbed, and therefore, these areas were excluded. Ports involved only a small number of observations (47), accounting for <15% of the total number. In addition, the highly degraded environmental conditions and the type of area management employed in ports exclude habitat conservation.

### Data

Our data came from the Mediterranean Elasmobranch Citizen Observation (MECO) project. The MECO is based on social media reports of people's encounters with sharks and rays. The project was established in Israel in 2014 following social media posts of encounters between divers and elasmobranchs (Barash et al., 2018). The project uses citizen science and social media to collect and make available knowledge about the occurrence, distribution, and seasonality of sharks and rays in the Mediterranean Sea. The project uses local shark and ray experts to manage a native‐language Facebook group that collects reports of photographed shark and ray encounters.

When people join the group, they receive an explanation of the group's goals and of how researchers will use what they report. By joining, they agree to the future use of the information they provide. Reports require a photo or a video, an approximate location, and the observation date, which must be within a month of accuracy. A trained MECO member reviews the information, and, in most cases, further information is requested, such as approximate length and weight, depth of guitarfish observed, number of individuals observed, and type of activity engaged in during the encounter (e.g., diving, swimming, angling). The direct request for further information from the researcher to the reporter creates an open dialogue that enables direct communication between the group participants and the researchers.

Members of MECO are local researchers who are experts on various aspects of shark and ray ecology and biology. Based on the image or video, the researcher identifies the fish as accurately as possible. If they can, experts add maturity and gestation data and sex based on the report's documentation. Since its establishment, MECO has expanded to 11 countries and 10 Facebook groups (Barash et al., 2018).

As of October 2022, the Israeli MECO data set we used contained 571 guitarfish observation records, dating from 2014 to October 2022, that included both species. We divided the observations of each species of guitarfish into 2 life stages, adult and juvenile, based on maturity size described in the literature: 60 cm for male and 70 cm for female common guitarfish and 100 cm for male and 110 cm for female blackchin guitarfish (Ebert & Dando, 2020). The size of the guitarfish was deduced by cross‐checking the observer assessment with an assessment by an expert in relation to objects of known size (i.e., fishing rod, human hand, shellfish, etc.). Another way we verified maturity in male guitarfish was based on whether claspers extended beyond the pelvic fin (Follesa & Carbonara, 2019). Adult guitarfish were separated into males and females based on the presence or absence of claspers. We discarded records with incomplete identification (i.e., species, life stage, and sex with the adult guitarfish) and those reported from commercial fishing, ports, or the authorities (i.e., the INPA). Only one observation per site per day per activity was used in the analyses. This left 393 observations divided into 6 categories: adult female blackchin guitarfish (69), adult male blackchin guitarfish (23), juvenile blackchin guitarfish (171), adult female common guitarfish (47), adult male common guitarfish (26), and juvenile common guitarfish (57). The 393 observations were reported by 233 different people, resulting in an average of 1.69 reports per person.

Based on the site location and the description of the sightings from social media posts, each observation was given a geographic reference point. In addition to locating the guitarfish's presence, we used the same observations to map human activities of 8 kinds. These were scuba diving, free diving, snorkeling, swimming, beach walking, beach angling, free dive spearfishing, and other fishing to accommodate for observations that were rare (e.g., angling from boats or kayaks). Our model considers these activities as conservation features for the analyses, regardless of whether the interaction reported was negative or positive. Because both guitarfish and human uses are mobile, we demarcated an area around each observation point to represent the estimated presence area for guitarfish (250 m for juveniles and 500 m for adults) and human activities (1000 m). Adding this area corrected location errors arising from a close, but likely inaccurate, reference point.

### Optimization modeling

To identify candidate protection areas for guitarfish conservation based on the encounters, we used Marxan. This software is commonly used for spatial conservation prioritization site selection (Janßen et al., 2019). It uses a heuristic optimization algorithm and simulated annealing to create a portfolio of planning units that when considered together meet specific conservation goals at the lowest possible cost (Ball et al., 2009). To create Marxan input files, we used QGIS 3.28.1 and the plugin Conservation Land‐Use Zoning software (CLUZ). The CLUZ was developed to assist in creating the Marxan input files. In addition, it automatically generates a gap analysis and allows for on‐screen conservation planning (Smith, 2019). The built‐in gap analysis allowed us to assess whether the area of the current MPAs and those planned meets the goals we defined for guitarfish habitat conservation (Table 1).

**TABLE 1:** Scenarios modeled with the decision‐support tool Marxan for identifying priority areas for conservation, including percent conservation goal of guitarfish habitat and human recreational use areas.

Scenario^*^	Guitarfish habitat (%)	Human‐use areas (%)
1	30	0
2	50	0
3	30	50
4	50	50

* Scenario definitions: 1, conservation of 30% of each guitarfish category habitat following the Convention on Biological Diversity (CBD) targets 3 and 4 of the COP15 protocol; 2, conservation of 50% of each guitarfish category habitat following post‐2020 biodiversity goal of the CBD; 3, conservation of 30% of each guitarfish category habitat and conservation of 50% of the different human recreational use areas; 4, conservation of 50% of each guitarfish category habitat and conservation of 50% of the different human recreational use areas.

Marxan is one of the most accepted decision‐support tools for conservation (Chen et al., 2022; Janßen et al., 2019). Its approach is based on the principle of complementarity, meaning selecting how planning units considered together in a particular configuration result in the highest level of biodiversity overall (Watts et al., 2017). Marxan has been used for designating protected areas in the terrestrial (Law et al., 2017; Smith et al., 2022) and marine environments (Delavenne et al., 2012; Holness et al., 2022; Markantonatou et al., 2021; Mazor et al., 2014). Its advantages include flexibility and the ability to analyze complex data (Ball et al., 2009). However, among its drawbacks is the need for large data sets that are sometimes hard to come by (Janßen et al., 2019). Though Marxan focuses on finding locations with the greatest richness in biodiversity that complement each other, it has been used for other purposes, such as identifying areas for offshore wind farms (see Göke et al., 2018). In our case, Marxan addresses human–marine wildlife encounter sites as points of interest.

The study area was within 2 km of the coast because our observation data were aggregated along the coastline. We had no observations that met the previously mentioned criteria farther offshore. We divided the planning area into 250‐m^2^ hexagons. Data on preexisting MPAs provided information on how much guitarfish habitat was protected. This information was obtained and processed from a layer provided by the INPA (https://www.parks.org.il/). Data on port and marina areas were taken from the Policy Document for Israel's Maritime Space (Israel Planning Administration, 2022).

We use 4 scenarios to analyze the conservation of guitarfish and their encounters with humans. The first 2 scenarios included only guitarfish conservation, and the other 2 included human uses and guitarfish conservation (Table 1). We assumed that guitarfish observations indicate the location of guitarfish habitat. The goal of 30% conservation of guitarfish habitat is based on international conventions of which Israel is a member—in particular, the Convention on Biological Diversity (CBD). The CBD targets 3 and 4 of the COP15 protocol aim to ensure that by 2030, at least 30% of terrestrial, inland waters and of coastal and marine areas are effectively conserved and that management actions are taken to protect threatened species (CBD, 2022). Our 50% guitarfish habitat conservation goal is reasonable considering that globally both guitarfish species are critically endangered. Biological and ecological knowledge about both species is lacking; therefore, conserving both at a higher level is precautionary. In addition, 50% area conservation was adopted by the CBD as a future goal of the post‐2020 biodiversity framework of the CBD (CBD, 2018). The other 2 scenarios combined the guitarfish habitat conservation goals with recreational use area conservation goals of 50%. The first scenario consisted of 30% conservation of each guitarfish category habitat and 50% conservation of each human activity area, and the second scenario consisted of 50% conservation of each guitarfish category habitat and 50% conservation of each human activity area (Table. 1)

For each scenario, we did 100 Marxan runs (each with one million iterations) based on gray and academic literature (Delavenne et al., 2012; Game & Grantham, 2008). Following a sensitivity analysis, the boundary length modifier, which controls the compactness of selected areas, was set to 0.2. We gave all conservation features the same level of importance; hence, we set the penalty factor, which accounts for the relative importance of different conservation features, to 10 for guitarfish (all categories) and for all activities (Game & Grantham, 2008).

## RESULTS

Adult male blackchin guitarfish had the smallest gap in conserved habitat, with 28.6% of their habitat already in an existing MPA, 1.4% short of the first and third scenarios’ 30% habitat protection goal (Table 1). Adult female common guitarfish and the adult female blackchin guitarfish habitat were also very close to the 30% habitat protection goal, with 25.5% and 24.5% of habitat in existing MPAs, respectively. The other 3 categories of guitarfish, juvenile common guitarfish, adult male common guitarfish, and juvenile blackchin guitarfish, had <30% of their habitat protected in existing MPAs (13.6%, 12.2%, and 8.7%, respectively). When looking at the INPA's plans for MPAs (https://www.parks.org.il/), the targets of 30% habitat conservation will be met. However, most of the categories, except for female common guitarfish, still fall short of the 50% habitat conservation target, especially the common guitarfish males and juvenile blackchin guitarfish. However, some of these MPAs have been included only because the INPA proposed their designation over a decade ago (Yahel & Engert, 2012). They have not yet completed all the necessary legal processes, which include final approval.

**FIGURE 3:**
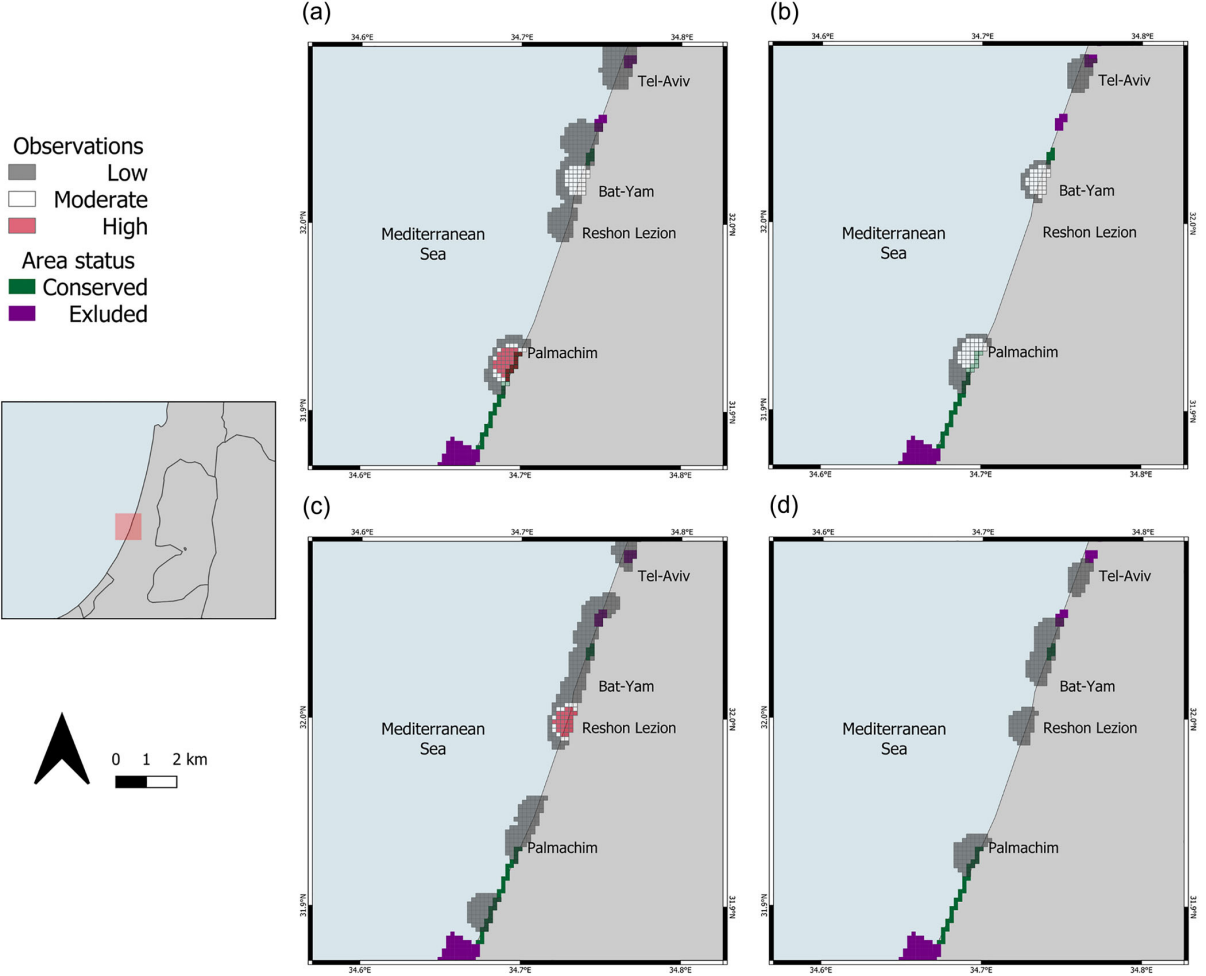
Distribution of (a) diving, (b) snorkeling, (c) angling, and (d) beach walking (percentage of observation from the same activity: low, ≤0.25%; moderate, 0.25–0.5%; high, >0.5%). The other activities included were not prominent in this area and are therefore not shown.

Another benefit of using CLUZ is that it allowed us to see the distribution of each conservation target separately and to overlay the Marxan outcomes so we could see where the main areas of each guitarfish category or human activity take place (Figures 2 & 3, respectively). Doing this enabled us to highlight areas for different conservation measures and, where the Marxan results still showed a gap for a specific conservation target, to identify areas that could help fill that gap.

The results showed that different activities occurred in different areas (Figure 3), that guitarfish of various life stages aggregated in certain areas, and that different categories of guitarfish were more frequently encountered during specific activities (Figures 2 & 3). For example, all 6 types were observed in the Palmachim area (Figure 2), yet only juveniles of both species were sighted in Rishon Lezion (Figure 2c,f). In the same Rishon Lezion area, there was also a high concentration of angling activity (Figure 3d).

**FIGURE 4:**
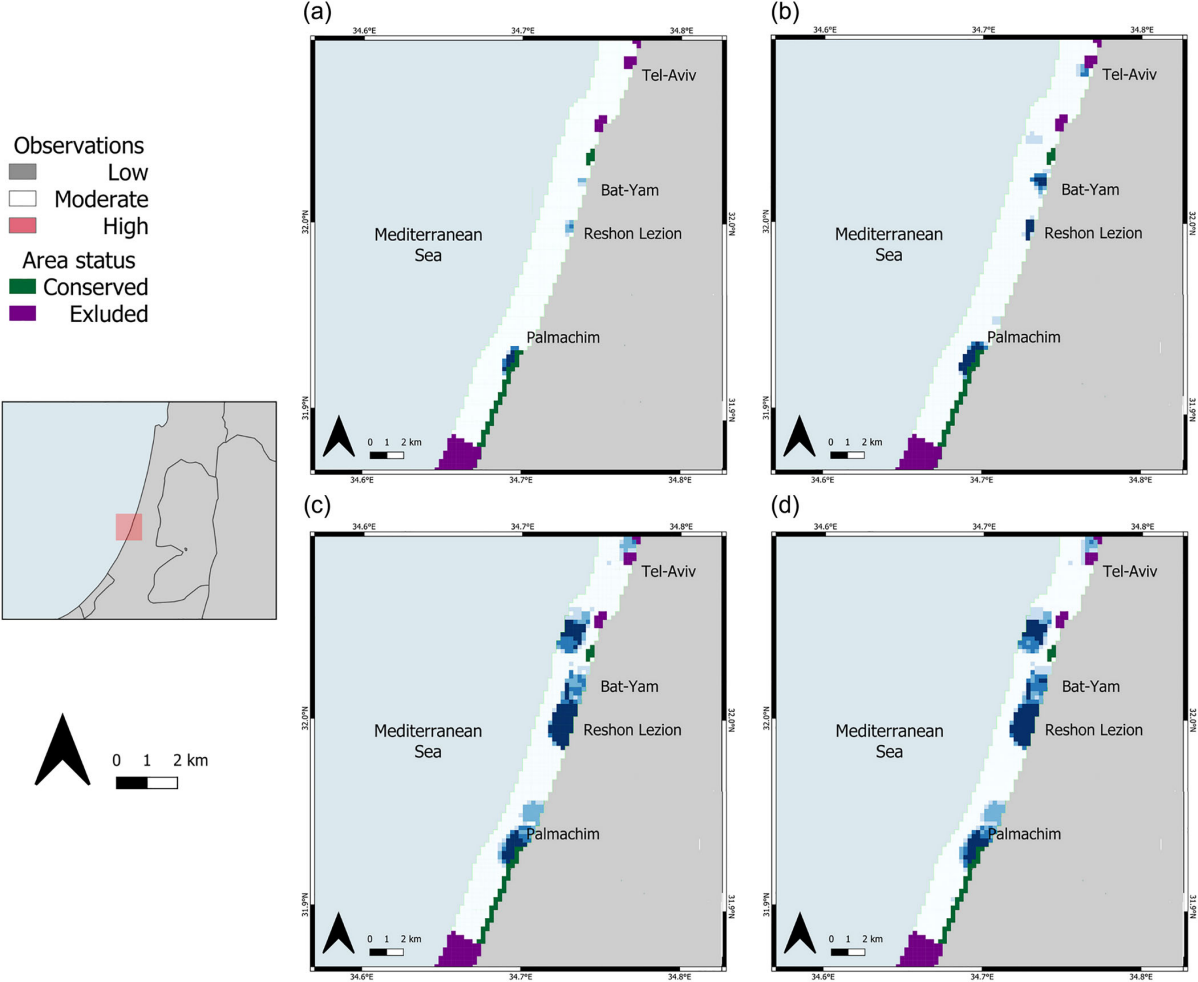
Marxan frequency of selection of priority conservation areas for 4 scenarios of guitarfish conservation and human recreational use areas: (a) 30% guitarfish habitat, (b) 50% guitarfish habitat, (c) 30% guitarfish habitat and 50% human activities, and (d) 50% guitarfish habitat and 50% human activities.

Most of the areas Marxan identified for conservation under all scenarios were from the central district to the south‐central district of Israel. No sites for protection were indicated in the northern district under any scenario. Three sites stood out as highly important for guitarfish conservation (Figure 4). These included areas proposed for expansion of the existing Palachim National Park and 2 sites next to the cities Reshon Lezion and Bat‐Yam south of Tel Aviv (Figure 1). Large areas were identified for protection when human uses were added to the targets (Figure 4).

## DISCUSSION

Considering human–marine wildlife encounters in marine planning is advantageous. These encounters can be positive and mutually enhancing—for wildlife because they generate interest in conservation and for human recreation because they supply interest and enjoyment. When interactions are negative, regulatory interventions can be applied through marine planning, thus ensuring more of an ecosystem‐based approach. Tracking, processing, and analyzing the encounters systematically is complex, which often results in important data being omitted from the marine planning processes. Our research provides a case study that makes methodological and practical contributions.

The gap analysis results indicated that conservation, as proposed by the INPA in 2012, will succeed in reaching the 30% goal but will fall short of the 50% goal. Some studies that looked at areas included in the proposed MPAs (Gdor and Evtah) showed different results. The Gdor MPA, where aggregations of blackchin guitarfish have been described (Chaikin et al., 2020), emerged as significant in our study for guitarfish conservation. By contrast, the Evtah MPA where a blackchin guitarfish nursery area was identified in the past (Azrieli, 2020) emerged as insignificant for the guitarfish conservation habitat in our study. This lack of data from some areas is one limitation of using information provided solely by citizen science. However, the MECO project does include observations from nearby areas.

Because activities undertaken by observers are not prohibited in Israel's MPAs, a question arises as to why there are so few data points in the Evtah MPA. Is there a problem with the method or did the declaration of the area as an MPA change recreational uses in this area? There is no apparent reason for this gap. Yet, without further research, the question cannot be answered. Regardless of the answers to these important questions, these MPAs can be added as guitarfish conservation areas and help reach the 50% goal for the 2 species.

Nursery areas and breeding aggregation sites play a crucial role in the life cycle and overall population dynamics of guitarfish; hence, protecting areas where different life stages occur is important (Hyde et al., 2022). This can be done by focusing on the protection of different habitat types (e.g., nursery areas where more juveniles are observed). Like other cartilaginous fish, guitarfish have a long reproductive cycle and produce fewer offspring than bony fish (Lteif et al., 2016; Newell, 2017). Protecting key locations for reproductive activities and the early stages of offspring development is essential for the species’ survival (Heupel et al., 2007). Though not the focus of this study, our results showed aggregations of different life stages, which can be used to identify areas with the potential of being nursery areas. Further research is needed to determine whether these areas meet the 3 criteria for nursery areas: higher density of juveniles than in other sites, presence of juveniles for an extended period, and multiple‐year presence of juveniles (Heupel et al., 2007).

Research performed on other elasmobranchs suggests that although a considerable emphasis has been given to the protection of nursery areas, the protection of neonates and juveniles may not contribute to the recovery of the populations as much as other life stages (Brewster‐Geisz & Miller, 2000; Kinney & Simpfendorfer, 2009). We treated all life stages similarly even though we noted that guitarfish of different life stage categories tended to aggregate in different locations (Figure 4). An emphasis on a particular life stage, once known, can be easily prioritized using our methodology (including Marxan).

The use of citizen science data collected by the MECO project allowed for a deeper understanding of the planning area with minimum intervention. The benefits of collecting information through citizen science have been described and widely debated in the literature (Cohn, 2008; Ellwood et al., 2017; Kosmala et al., 2016; McAteer & Flannery, 2022; Silvertown, 2009). Among lesser‐acknowledged advantages of citizen science based on social media is that sharing observations is an additional source of enjoyment. This data collection method is of high value in the planning realm. The method could help bring to the table sectors that were not previously represented in the planning processes and sometimes felt overlooked (Arlinghaus et al., 2019; Potts et al., 2020). Furthermore, it could help identify various use sectors and thus help improve EBM for marine planning. Involving stakeholders is an essential part of the marine planning process (Ehler & Douvere, 2009b; Long et al., 2015), yet not all are recognized and acknowledged. In addition, the dialogue between the experts and citizen observers allows for a 2‐way transfer of information. It encourages, to some extent, the integration of science, policy, and public participation (Adler‏ et al., 2020), a key component of successful EBM (Gibble et al., 2020; Long et al., 2015). Using information about the activities can encourage cooperation and compliance with planning outcomes and diffuse feelings of frustration.

Analyses of the different types of activities showed that areas were used for different purposes. Activities, such as diving and snorkeling, occurred in similar areas. In contrast, angling occurred in different areas (Figure 3). This spatial separation of recreational uses adds more layers for consideration when planning habitat protection. It allows the implementation of different conservation mechanisms as needed. Cross‐referencing human recreational use data with wildlife habitat data allows for accurate planning for both guitarfish and human recreational activities. For example, a city with a beach full of anglers can consider adding designated areas suitable for angling, thus supporting recreational fishing. A municipality can prohibit fishing that could harm divers and swimmers recreating on the same beach and thereby prevent conflicts between people. The Israeli marine plan suggests designating fishing areas around existing marine structures and further developing dedicated piers for angling (Israel Planning Administration, 2022). Our work can indicate areas for these piers.

As mentioned, we did not define which activities constituted positive interactions and which were negative. For example, diving encounters have the potential to be both positive and negative (Gallagher & Huveneers, 2018; Trave et al., 2017). Yet, by utilizing our method and incorporating data on human–wildlife interactions, measures can be taken to lower the chances of a negative encounter and increase the chances of positive encounters that promote conservation. For example, catch and release of guitarfish as bycatch by anglers may cause damage or even death if the individual is badly handled. Based on our results, areas frequented by anglers can be identified and educational programs can be planned for these areas for best practice of guitarfish handling and release.

Our use of citizen science data has limitations and biases. For example, an angler citizen observer can photograph a guitarfish up close and from several angles, which allows experts to identify the guitarfish more accurately. Moreover, fishing is not affected by water turbidity and sea conditions in contrast to other uses, thus creating a bias toward conservation based on fishing interactions. To try and counter some of these biases, one can map the uses without identifying the guitarfish to the species and life stage levels. Another bias is toward more observations posted from accessible beaches that are near settlements and that are easily accessible to the public. However, knowledge about the approximate time of year to conduct complementary conventional scientific surveys on these less accessible beaches could be gleaned from the initial (biased) observations.

We did not use data on commercial fishing of guitarfish, which is one of the main threats to both common and blackchin guitarfish (Jabado et al., 2021; Kyne & Jabado, 2019). Although there is little to no targeted fishing of the 2 guitarfish species in Israel because it is prohibited (Ministry of Justice, 2019), bycatch of guitarfish occurs, but it seems to be relatively limited. However, there were no official data on guitarfish as bycatch. Although the use of fisheries data of all types is well accepted and is frequently employed in conservation prioritization (Chen et al., 2022; Delavenne et al., 2012; Markantonatou et al. et al., 2021), in this study, we offer a new source of data and a new method to be incorporated into conservation prioritization modeling.

We did not use data on the number of individual guitarfish in every sighting. Such information can highlight hotspots of guitarfish presence. However, the count of individuals can contain errors, especially for fast‐moving, highly camouflaged targets, such as guitarfish, in highly turbid waters. In addition, count data can cause a shift in importance to a certain area based on a particularly rich observation, which in turn causes other important areas to be ignored. These rich observations came from activities such as diving, snorkeling, and beach walking. For example, there was one observation of over 100 blackchin guitarfish juveniles at one site (Ma'agan Michael). Giving weight to the number of individuals in each observation would have shifted our results so that this site would have been the only site to protect 30% and even 50% of juvenile blackchin guitarfish areas. Yet, despite not weighing the number of individuals in this single observation, there were multiple observations from Ma'agan Michael, and it emerged as a significant area for juvenile blackchin guitarfish. The significance of Ma'agan Michael is also supported by Azrieli (2020), who identified this area as a nursery for blackchin guitarfish.

Despite some biases, it is important to understand where the human–wildlife interactions generally take place and address them within the planning processes. Such understanding can lead to decisions about how the area can be designated so that the encounters encourage nature conservation and reduce negative encounters. The fact that large areas were identified for protection when human uses were added to the targets (Figure 4) suggests the importance of referring to human leisure activities as a conservation goal. This is especially true considering that an area not defined as an MPA, particularly those close to large urban areas, could undergo massive development, producing harmful activities not always considered during the marine planning process.

Our case study showed that including human–marine wildlife interactions indicated new areas to consider when approaching conservation planning, thus adding a new dimension to conservation planning that supports EBM. Making nature accessible to people while maintaining the use of marine areas without unnecessary prohibition or restriction of recreational activities can defuse any resistance toward protection while enhancing marine wildlife conservation.
